# Prevalence of *S. aureus* and/or MRSA in hospitalized patients with diabetic foot and establishment of LAMP methods for rapid detection of the SCCmec gene

**DOI:** 10.1186/s12866-024-03196-6

**Published:** 2024-01-26

**Authors:** Chunxia Qi, Xiangrong Luo, Jiali Huang, Danli Kong, Yali Zhang, Mengchen Zou, Hao Zhou

**Affiliations:** 1 Department of Hospital Infection Management, NanFang Hospital of Southern Medical University, Guangzhou, Guangdong 510510 P.R. China; 2 Department of Endocrinology and Metabolism, Nanfang Hospital of Southern Medical University, Guangzhou, Guangdong 510510 P.R. China; 3 Department of Epidemiology and Medical Statistics School of Public Health, Guangdong Medical University, Dongguan, Guangdong 524023 P.R. China; 4 Department of Hospital Infection Management, ZhuJiang Hospital of Southern Medical University, Guangzhou, Guangdong 510280 P.R. China

**Keywords:** MRSA, Diabetes, Infection, Prognosis, LAMP, Subtype, SCCmec

## Abstract

**Background:**

Patients with diabetic feet are prone to be infected due to the impaired immune system. However, the prognostic outcome of different microbial infections remains controversial. Identification and rapid screening of the pathogenic microorganisms that pose the greatest threat to the prognosis of patients with diabetic foot infections (DFIs) is critical.

**Methods:**

Clinical data were statistically analyzed, which were obtained from 522 patients with DFIs, including pathogenic bacterial culture results and treatment outcomes at the last return visit. In addition, a loop-mediated isothermal amplification (LAMP) detection method was developed to identify the prevalent subtype of methicillin-resistant *Staphylococcus aureus* (MRSA) in DFIs patients. This study was approved by the Ethics Committee of Nanfang Hospital (NFEC-202012-K6) and registered on ClinicalTrials.gov (NCT04916457) on June 1, 2021.

**Results:**

We found that the proportion of patients with infections of *Staphylococcus aureus* (*S. aureus*) and MRSA was 27.7% (145/522) and 33.7% (49/145), respectively. Additionally, the incidence of osteomyelitis was 46.9% (23/49) and amputation/disability was 40.8% (20/49) in patients with MRSA infection, which were significantly higher compared to patients with other types of bacterial infections such as methicillin-susceptible *Staphylococcus aureus* (MSSA). Notably, we demonstrated that the main prevalent subtype of MRSA in DFIs patients in our hospital was Staphylococcal chromosomal cassettes mec (SCCmec) type II. In addition, it only takes 1.5 h to complete the entire experimental procedure in this LAMP assay, providing high sensitivity (100%) and specificity (77.8%) in hospitalized patients with DFIs.

**Conclusions:**

We demonstrated there is a very high rate of MRSA isolation in patients with DFIs and revealed that patients infected with MRSA are at a higher risk of developing osteomyelitis, and amputation or disability. Importantly, we have developed a method for quickly screening newly admitted patients for MRSA.

**Supplementary Information:**

The online version contains supplementary material available at 10.1186/s12866-024-03196-6.

## Introduction

Diabetic foot ulcers (DFUs) are a common chronic complication observed in diabetic patients and often lead to secondary infection, deep tissue destruction, and sepsis [1]. Patients with diabetes have a 19–34% chance of developing DFUs, with a recurrence rate of up to 50–70% within 5 years [2]. Persistently elevated blood glucose levels lead to immune dysfunction, resulting in impaired leukocyte activity and complement function. This, in turn, promotes the development of invasive tissue infections, commonly referred to as diabetic foot infections (DFIs). A wide variety of pathogenic microorganisms have been detected in patients with DFIs, including staphylococci, streptococci, enterococci, and enterobacteria [3]. The emergence of antibiotic-resistant bacteria has significantly increased the challenge of managing DFIs. Without prompt treatment, these bacteria can infect other tissues and potentially result in the need for amputation [4].

Methicillin-resistant *Staphylococcus aureus* (MRSA), the most common gram-positive bacterium isolated from DFIs patients, can develop resistance to all β-lactam agents due to the presence of the acquired mecA gene. This gene produces a penicillin binding protein (PBP2a) with low affinity for β-lactam antibiotics [5]. Identification of patients with MRSA infection is essential for effective treatment of DFIs [6]. It usually takes 3–5 days to detect MRSA using conventional culture methods in the clinic, resulting in the best time for treatment being missed. Recently, multiple rapid detection methods have been developed to identify MRSA by targeting the mecA gene and the *S. aureus*-specific femA/nuc gene [7, 8]. Considering the high percentage of coagulase-negative methicillin-resistant staphylococci (MRSCN) carrying the mecA gene that may be present in specimens from patients with diabetic foot [9], these methods may not be able to distinguish MRSA in the presence of MRSCN, leading to the incorrect use of vancomycin for treating MRSA.

Staphylococcal chromosomal cassettes mec (SCCmec) is a mobile genetic element that carries a mec gene complex encoding the PBP2a protein and a ccr gene complex encoding recombinases [10]. The SCCmec type is an important and specific characteristic used to identify MRSA clones in conjunction with the *S. aureus* chromosome genome. To date, 13 types of SCCmec have been identified, labeled as I to XIII [11]. The SCCmec epidemic subtypes of MRSA vary from region to region, but there is typically only one predominant subtype in a region, such as type II, III, or IV [12]. Loop-mediated isothermal amplification (LAMP) can be used to produce up to 10^9–10^10 copies of amplified DNA within an hour at a constant temperature. The results can be observed through fluorescence, colorimetric, or precipitate methods [13].

Previous studies have provided limited information on evaluating the impact of MRSA infection on long-term treatment outcomes and the length of hospitalization in DFIs patients. Additionally, a rapid method for detecting MRSA in DFIs, which excludes interference from MRSCN, has not been reported. In this study, we attempted to analyze the proportion of *S. aureus* infections, specifically MRSA, among DFIs patients in our hospital. We also sought to emphasize the importance of timely detection and treatment of MRSA infection by investigating the correlation between MRSA infection and adverse outcomes. In addition, we aimed to identify the predominant subtypes of SCCmec among the MRSA strains isolated from patients with DFIs. We also attempted to develop a rapid and direct screening tool with high specificity and sensitivity for newly admitted patients, which can be used to assist hospitals in providing accurate medication on the day of admission and help infection control departments prevent the occurrence of nosocomial outbreaks of MRSA infections.

## Results

### Bacterial species isolated from patients with DFIs

Retrospective analysis of the microbiological information from 522 hospitalized patients with DFIs revealed that there was a slightly higher proportion of patients with single infections (277 cases) compared to patients with mixed infections (245 cases) (53.1% vs. 46.9%). In patients with single bacterial infections, the detection rate of *S. aureus*, including MSSA and MRSA, was significantly higher than that of other types of bacteria, reaching up to 30.3% (84/277). This was followed by coagulase-negative Staphylococcus (19.1%, 53/277) and Enterobacter (22.4%, 62/277).

Moreover, we found that *S. aureus* was isolated in 145 of the DFIs patients (27.8%, 145/522), including 84 cases of single infection (57.9%, 84/145) and 61 cases of mixed infection (42.1%, 61/145). We also revealed that the proportion of patients with MRSA infection was as high as 33.8% (49/145), with up to 63.3% (31/49) of those patients having a single MRSA infection (Table 1).

**Table 1:** Clinical data of 522 patients with DFIs

Type		Group	Cases	Sex		Age		
				Male	Female	30–49 years	50–69 years	70–89 years
S. *aureus* infection (145 cases)	Single Infection (84 cases)	Only MSSA	53	37	16	12	29	12
		**Only MRSA**	**31**	**23**	**8**	**4**	**23**	**4**
	Mixed Infection (61 cases)	Multiple bacteria were co-isolated with MSSA	43	33	10	4	31	8
		**Multiple bacteria were co-isolated with MRSA**	**18**	**11**	**7**	**4**	**5**	**9**
Other types of bacterial infection (377 cases)	Single Infection (193 cases)	Only coagulase-negative Staphylococcus	53	33	20	6	35	12
		Only Enterococcus	25	18	7	2	17	6
		Only Streptococcus	19	17	2	6	12	1
		Only Enterobacter	62	38	24	11	36	15
		Only non-fermentative bacteria	19	13	6	1	10	8
		Only Candida	15	9	6	1	10	4
	Mixed Infection (184 cases)	Multiple bacteria without MSSA/MRSA	184	120	64	17	107	60

No significant differences were found in sex and age among the infection groups (*P* ≥ 0.05)

The bolded characters represent information about patients who were infected with MRSA

*Abbreviations*: *DFIs* Diabetic Foot Infections, *MSSA* Methicillin-Susceptible *Staphylococcus Aureus*, *MRSA* Methicillin-Resistant *Staphylococcus Aureus*

### Association between worse outcomes and MRSA infection in DFIs patients

We performed a statistical analysis of the correlation between MRSA infection and osteomyelitis, amputation/disability, as well as length of hospital stay. Our findings revealed that patients with a single MRSA infection had the highest rates of osteomyelitis and amputation/disability (48.4%, 48.2%). This was closely followed by patients with mixed infections that included MRSA (44.4%, 33.3%). These proportions were significantly higher than those observed in the other groups, including the MSSA mono-infection group (20.8%, 20.8%) and the mixed infection group (14.0%, 14.0%). Notably, the incidence of osteomyelitis and amputation/disability was only 5.7% and 11.3% in patients with coagulase-negative staphylococcal infection, respectively. Additionally, both rates were only 25.8% in patients with Enterobacter infections (Table 2).

**Table 2:** Statistical analysis of treatment outcomes in 522 patients with DFIs

Type	Group	Cases	Osteomyelitis	Amputation/Disability	Length of hospital stay				
					Median	P25	P75	IQR	Range
145 cases of *S. aureus* infection	Only MSSA	53	11 (20.8%)	11 (20.8%)	11	8	15.5	7.5	5–37
	**Only MRSA**	**31**	**15 (48.4%)**	**14 (45.2%)**	**16**	**12**	**22**	**10**	**5–45**
	Multiple bacteria were co-isolated with MSSA	43	6 (14.0%)	6 (14.0%)	13	8	21	13	5–42
	**Multiple bacteria were co-isolated with MRSA**	**18**	**8 (44.4%)**	**6 (33.3%)**	**18.5**	**10**	**28.25**	**18.25**	**6–43**
377 cases of other types of bacterial infection	only coagulase-negative Staphylococcus	53	3 (5.7%)	6 (11.3%)	12	9	17.5	8.5	3–46
	Only Enterococcus	25	5 (20.0%)	5 (20.0%)	14	5	49	44	9–22.5
	Only Streptococcus	19	4 (21.1%)	3 (15.8%)	12	8	20	12	3–29
	Only Enterobacter	62	16 (25.8%)	16 (25.8%)	14	9	22	13	4–40
	Only non-fermentative bacteria	19	3 (15.8%)	2 (10.5%)	18	11	28	17	4–63
	Only Candida	15	2 (13.3%)	2 (13.3%)	11	8	21	13	6–67
	Multiple bacteria without MSSA/MRSA	184	37 (22.1%)	38 (20.7%)	16	10.25	22.75	12.5	1–69

Significant differences were observed in the rates of Osteomyelitis, Amputation, and hospitalization days among the infection groups (*P* < 0.05)

Data is presented as n (%). The bolded characters represent information about patients who were infected with MRSA

*Abbreviations*: *DFIs* Diabetic Foot Infections, *MSSA* Methicillin-Susceptible Staphylococcus Aureus, *MRSA* Methicillin-Resistant Staphylococcus Aureus, *IQR* (InterQuartile Range)

Next, we analyzed the hospital stay of patients with a single infection and found that patients with non-fermentative bacterial infections had the highest number of days hospitalized, which was up to 18 days (IQR, 17). Patients with MRSA infections had a length of stay of 16 days (IQR, 10), which was significantly higher than that of 11 days (IQR, 7.5) observed in the MSSA infection group (Table 2).

### Evaluation of the sensitivity and specificity of femA and mecA primers in strains

**Fig. 1:**
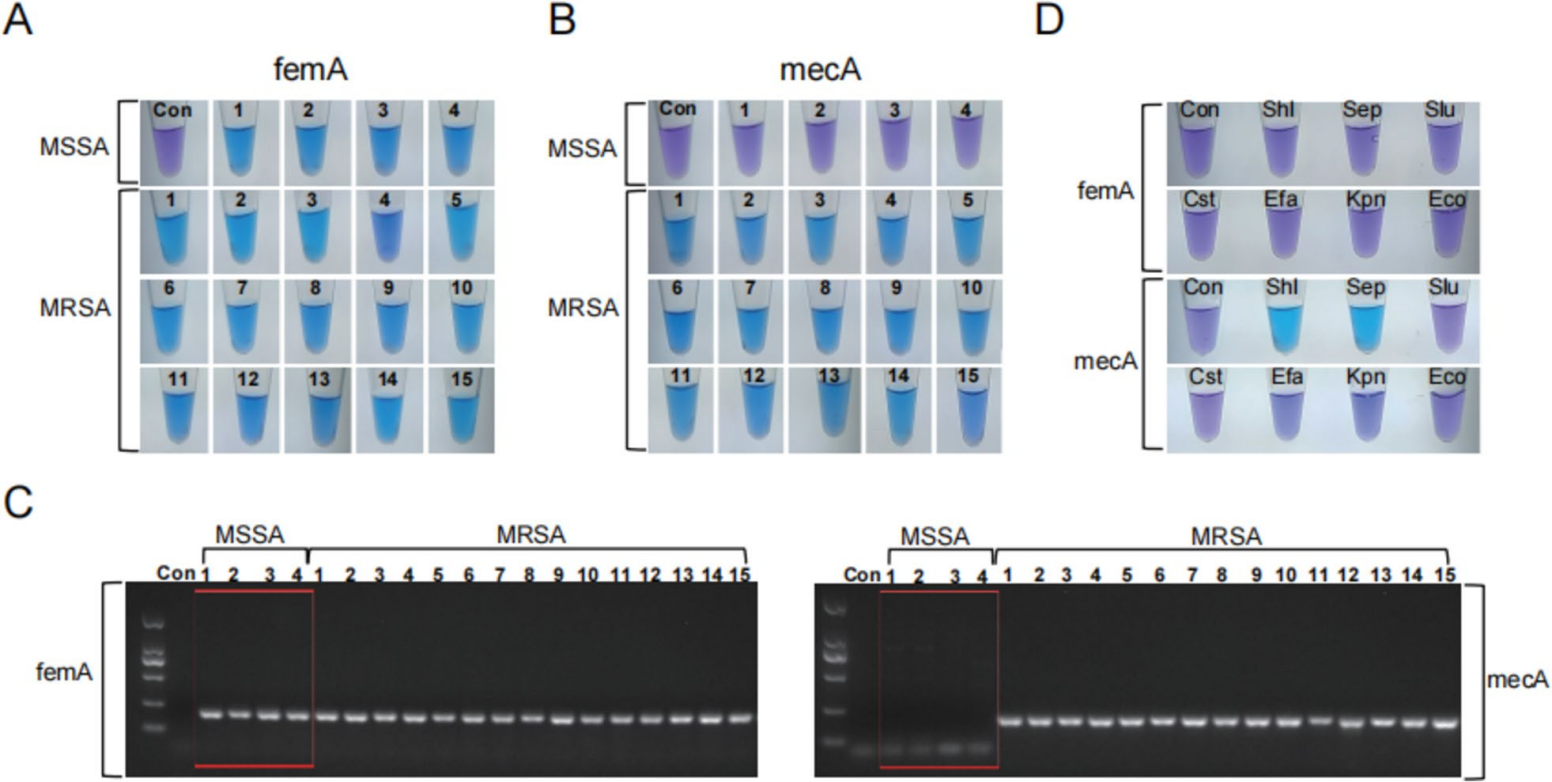
Sensitivity and specificity of the LAMP assay based on femA and mecA in strains. **A** 4 strains of MSSA and 15 strains of MRSA were tested using the LAMP method for the femA gene. All 19 strains were obtained from the hospital laboratory and analyzed using mass spectrometry. The drug resistance of the MRSA strains was determined through drug sensitivity testing. **B** 4 strains of MSSA and 15 strains of MRSA were tested using the LAMP method to detect the mecA gene. **C** PCR amplification of femA and mecA for 4 MSSA strains and 15 MRSA strains. **D** LAMP assays for the presence of femA and mecA in seven bacterial species. Abbreviations: Con, control group; *Shl*, *Staphylococcus haemolyticus; Sep, Staphylococcus epidermidis; Slu, Staphylococcus lugdunensis; Cst, Corynebacterium striatum; Efa, Enterobacter faecalis; Kpn, Klebsiella pneumoniae; Eco, Escherichia coli*

To evaluate the practical effectiveness of the LAMP method in identifying MRSA infections in DFIs patients, we designed two specific primers targeting femA and mecA genes. These primers were used to identify 4 MSSA strains and 12 MRSA strains that were isolated from clinical patients. It was found that this method could be used to identify MRSA strains with 100% sensitivity (Fig. 1A, B) and was in complete agreement with the PCR results (Fig. 1C).

**Fig. 2:**
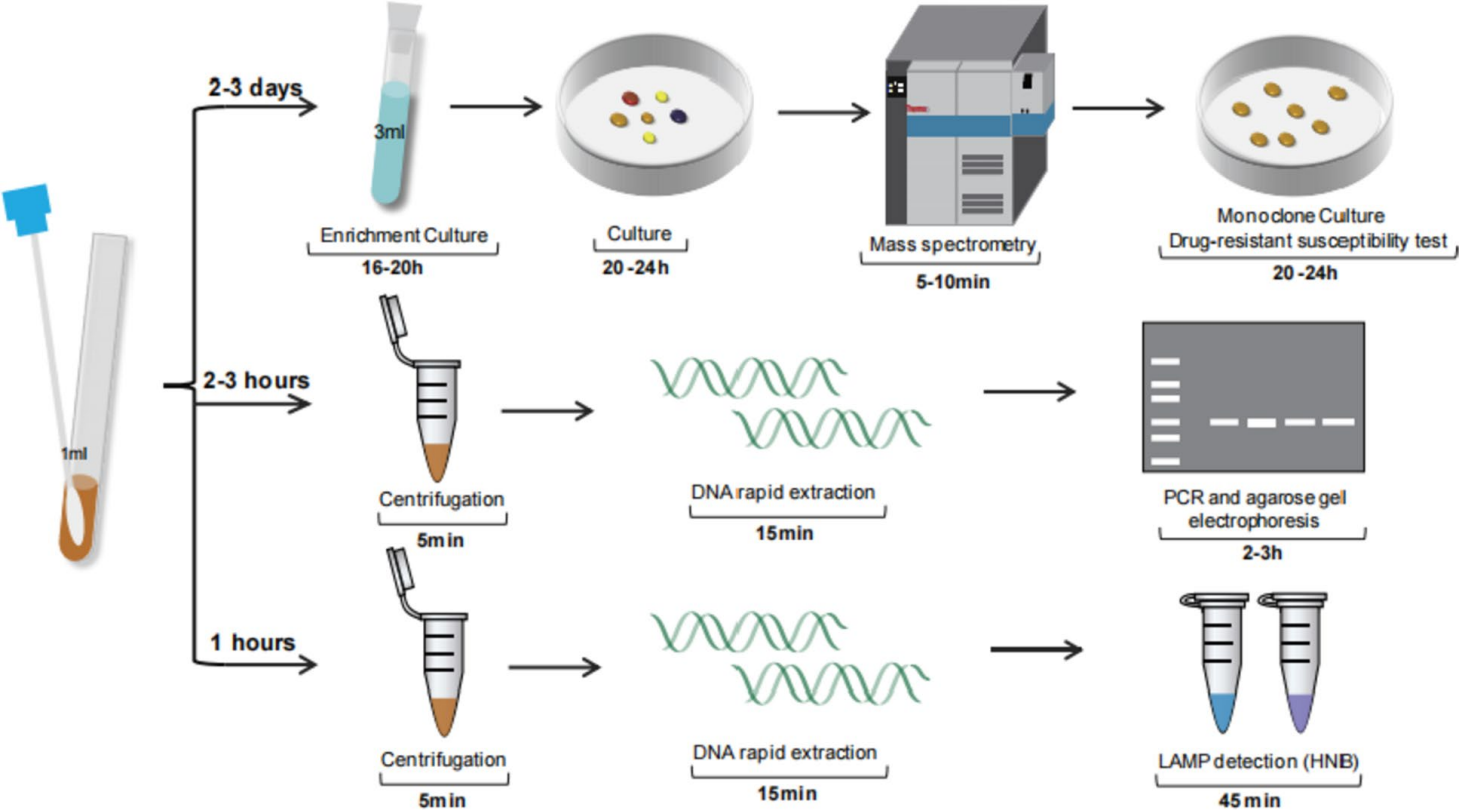
Schematic diagram illustrating the workflow of the three MRSA assays. The process includes monoclonal selection and drug sensitivity assay after enriching the culture of MRSA. It also involves PCR amplification and LAMP analysis system based on SCCmec element

Considering the complexity of bacterial species in clinical specimens, we selected seven bacteria commonly found in DFIs patients for further analysis. The results demonstrated that this method could effectively distinguish MRSA strains from these bacteria with 100% specificity (Fig. 1D). Notably, the LAMP detection used in this experiment can shorten the entire experimental cycle to less than 1 h (Fig. 2).

### Application of LAMP femA primers in the screening of *S. aureus* in DFIs patients

**Fig. 3:**
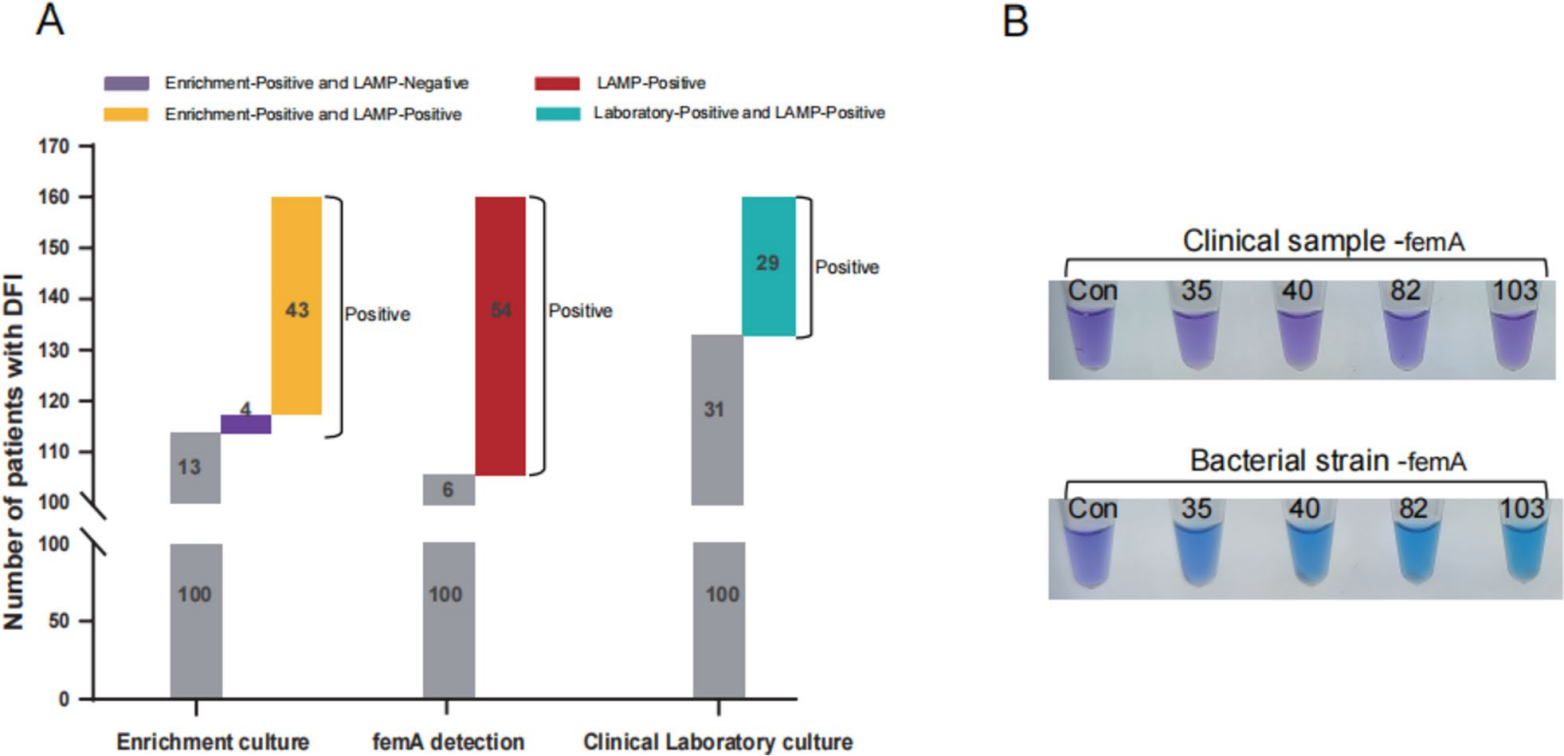
Determination of the cases of S. aureus infection in 160 clinical samples from patients with diabetes foot. **A** Comparing three methods for identifying the number of patients with S. aureus infection. Of the 160 clinical samples collected, S. aureus was detected in 47 samples using the enrichment culture method, in 54 samples using the LAMP assay technique, and in 29 samples using the clinical hospital laboratory culture method. **B** S. aureus strains in four clinical samples were analyzed using the LAMP assay targeting the femA gene. These four clinical samples could only be identified S. aureus in enrichment culture, while the results were negative in the LAMP test and clinical laboratory culture

We collected tissues from the foot wounds of 160 hospitalized DFIs patients and screened all samples for *S. aureus* using the LAMP femA primers method. Using conventional culture results from the clinical laboratory (29 cases of *S. aureus*) as a reference, we discovered that the enrichment culture of 47 cases of *S. aureus* yielded significantly more positive results than the conventional culture. The LAMP femA primers test of 54 cases of *S. aureus* showed the highest rate of positive tests (Fig. 3A).

Interestingly, we found that four clinical samples tested positive for *S. aureus* only in the enrichment culture assay, while they were tested negative in both the conventional culture and femA primer assays. To determine if the negative LAMP detection results were caused by mutations in the femA gene, we conducted further analysis on the *S. aureus* strains isolated from the four clinical samples that showed positive results. Our result suggests that the false negatives observed in the femA primer assay in clinical samples may have resulted from using a low concentration of DNA template for amplification (Fig. 3B).

### Application of SCCmec type II primers in the identification of MRSA in DFIs patients

**Fig. 4:**
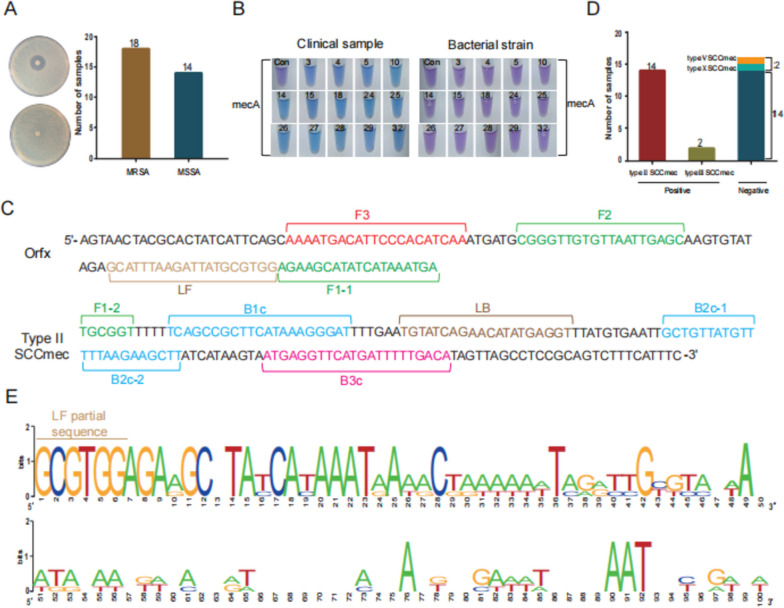
Analysis of the SCCmec subtype of MRSA isolated from clinical samples of patients with diabetic foot infections. **A** Antimicrobial sensitivity testing was conducted on 32 clinical samples that were tested positive for both femA and mecA in the LAMP detection. **B** Analysis of 14 S. aureus strains isolated from clinical samples was conducted using the LAMP method targeting the mecA gene. These clinical samples were found to be positive for mecA and negative for drug sensitivity testing. **C** Schematic diagram of LAMP primers for SCCmec type II. The target of this primer is located at the 5' end of the orfx gene of S. aureus and at the 3' end of the SCCmec element. FIP (forward inner primer): F1c + F2, BIP (backward inner primer): B1c + B2. F1c: F1 complementary strand, B1c: B1 complementary strand. **D** Analysis of 32 clinical samples that were tested positive for both femA and mecA using LAMP primers targeting type II and type III SCCmec components. **E** Comparison of the gene sequences of two other subtypes of MRSA strains with type II SCCmec. The gene sequences of these two strains were obtained through high-throughput sequencing

We evaluated 160 samples using both femA and mecA primers and screened 32 samples tested positive for both genes. To further confirm the presence of MRSA in these 32 samples, we isolated and cultured all *S. aureus* strains and performed drug susceptibility testing. The results showed that only 18 of these samples contained MRSA, while the remaining 14 contained MSSA (Fig. 4A, B).

To specifically identify MRSA infection in DFIs patients, we designed three pairs of LAMP primers targeting SCCmec I-III, which are prevalent subtypes of hospital-acquired MRSA. We then analyzed 18 clinical samples with MRSA infections. We found that the predominant subtype of MRSA in our hospital was SCCmec II, accounting for 77.8% (14/18) of cases, while the SCCmec III subtype had a rate of 11.1% (2/18). The other two strains were not subtyped (Fig. 4C, D). Further, we analyzed the subtypes of these two MRSA strains using high-throughput sequencing. The results showed that one strain was SCCmec V and the other was SCCmec X. Their gene sequences were completely different from the sequence of SCCmec II (Fig. 4E).

## Discussion

Patients with DFIs typically experience local trauma and microangiopathy. There is an estimated 15% risk that local tissue ulcers will progress to osteomyelitis, a condition that can result in amputation in severe cases. The mortality rate increases to 60% within five years after lower limb amputation [14]. Large-sample analysis of microorganisms isolated from DFIs patients in China revealed that 20% of the samples contained drug-resistant bacteria, and the proportion of MRSA was as high as 30.4% [15]. There is still controversy regarding whether the treatment outcomes of DFIs patients with MRSA are worse than those of patients infected with other pathogens, such as MSSA [16, 17]. In this study, we found that the rate of *S. aureus* isolation in patients with DFIs was as high as 27.8%, with MRSA accounting for 33.8%. Moreover, the rates of osteomyelitis and amputation/disability were significantly higher in patients with either single or mixed MRSA infections compared to patients with other types of bacterial infections, including MSSA. The ineffectiveness of antibiotic drugs was more common in patients with MRSA infections than in those with other bacterial infections, which may lead to exacerbation of the patient’s condition [18]. Therefore, rapid MRSA detection for diabetic foot patients can facilitate the prompt initiation of targeted treatment, thereby reducing the occurrence of osteomyelitis and the need for amputation or disability. This is crucial for the successful management of DFIs.

Diabetic foot patients with MRSA detection usually have a longer mean wound evolution and delayed healing [19]. We found that DFIs patients infected by a single non-fermentative bacteria had the longest hospital stay, with a median of 18 days. According to epidemiological analysis, the high rate of antimicrobial resistance associated with non-fermentative bacteria detected in hospitalized patients leads to a limited availability of effective oral antimicrobial drugs. Infections with these bacteria can only be treated by intravenous administration of antimicrobial drugs, which further prolongs the duration of hospitalization for patients [20, 21]. Importantly, the median length of hospital stay of DFIs patients with a single MRSA infection was second only to that of patients infected with non-fermentative bacteria. The number of hospitalization days was also much higher in patients with isolated MRSA than in patients without detectable MRSA, including those with mixed MSSA infection. This indicates that MRSA infection in patients with DFIs prolongs their hospital stay and can lead to various adverse consequences.

It has been reported that diabetic patients seem to be more frequently infected with *S. aureus*, and excess glucose levels in these patients considerably enhance *S. aureus* virulence potential, resulting in worse infection outcomes [22]. At present, the conventional methods for identifying MRSA include drug sensitivity testing and PBP2a latex agglutination testing. However, both methods require purified strains as the target, and these strains must be identified as *S. aureus* through mass spectrometry. Although they exhibit good sensitivity and specificity, these experiments usually take 1–2 days to complete [23, 24]. Therefore, the rapid identification of *S. aureus* in newly admitted patients with diabetic foot is essential to alleviate the patient’s condition. Here, we analyzed 160 trauma tissue samples from DFIs patients using the LAMP method with femA primers specific for *S. aureus*. Our analysis revealed a high isolation rate of 33.8% for *S. aureus* in the samples, which was not only higher than that obtained in the *S. aureus*-specific enrichment culture results (29.4%), but also significantly higher than that obtained in the conventional culture results (15.6%) used in the laboratory department. This finding reduces the risk of missed detection. Laakso, M evaluated data on pathogenic bacteria cultured from 405 patients who were hospitalized for DFIs and found that the most common bacteria in both superficial and deep samples was *Staphylococcus aureus*, with an incidence of 36.9% [25], which is consistent with the results of our experiment. In diabetic foot patients, the risk of recurrent DFIs is 2-fold higher in patients with multidrug-resistant bacterial (MDRO) infections compared to non-MDRO-infected patients [26]. Additionally, the majority of MDRO infections in diabetic foot patients are caused by MRSA.

Diagnosing MRSA infection as soon as possible can effectively help clinicians shift from empirical treatment to targeted therapy and shorten the treatment cycle for patients. Chen, C. applied the LAMP method with primers targeting mecA and nuc to detect 128 MRSA strains isolated from clinical samples and showed that the method was as effective as the PCR method [27]. In this study, we found that the LAMP method targeting the femA and mecA genes had a specificity and sensitivity of 100% in identifying isolated MRSA strains. According to our statistical results, multiple bacteria were isolated from 46.9% of DFIs patients, including a variety of skin-colonizing bacteria, the most common bacteria isolated was MRSCN, which carries a high percentage of the mecA gene. Here, we found that when using femA and mecA primers for analyzing MRSA infections in clinical samples from DFIs patients, this method produced a high false positive rate (43.8%, 14/32). This confirms that this strategy could not be used to distinguish MRSA infection in patients with polymicrobial infections. Therefore, this method can only effectively be used to identify* S. aureus* infection in DFIs patients but it cannot be used to determine whether the infection is due to MSSA or MRSA.

Nascimento, L. obtained 17 MRSA isolates from 34 DFIs patients, and the molecular results showed that 80% of the MRSA isolates carried SCCmec type I, while the remaining 20% were SCCmec type V [28]. However, several studies have demonstrated that the SCCmec IV subtype of MRSA is the most prevalent in patients with DFIs [29, 30]. Here, we found that the SCCmec II subtype was the predominant subtype of MRSA in DFIs patients in our hospital, accounting for 77.8% of cases. In contrast, the proportion of SCCmec III was only 11.1%. These findings suggest that the prevalence of MRSA subtypes varies across different regions. When utilizing the LAMP method for the rapid identification of the SCCmec gene in other hospitals or regions, it is essential to first analyze the predominant subtypes of MRSA.

In our hospital, this LAMP detection method targeting the SCCmec type II gene sequence can assist clinical doctors in conducting routine MRSA screening for newly admitted DFIS patients. It only takes about an hour to complete the entire experiment, and the results will enable rapid and accurate selection of the appropriate antibiotic medication for the patient on their first day of admission. At the same time, it also helps the department to promptly isolate hospitalized patients who test positive for MRSA and prepare for a range of infection control measures, including disinfection and protection. For clinical staff, the entire LAMP assay process can be conducted in-house within the clinical department. This method is cost-effective as it requires minimal investment in reaction equipment, detection equipment, and reagents. This assay has high specificity and can rapidly identify most MRSA infections. It enables accurate diagnosis, medication, and reduces the occurrence of negative side effects and treatment failures.

## Conclusion

Here, we found that MRSA infections accounted for 33.7% of all *S. aureus* infections in DFIs patients, and the incidence of both osteomyelitis and amputation was much higher in patients with MRSA infections than in patients with other types of bacterial infections, including MSSA infections. In addition, we have developed a LAMP detection method that can be used to identify the prevalent subtype SCCmec type II of MRSA among DFIs patients in our hospital. This method can be completed within 1.5 h and does not require the use of special instruments or specialized laboratory technicians. It is suitable for screening patients admitted to the hospital. However, this study was limited to patients in our hospital, and there may be regional differences in the prevalent subtypes of MRSA. Therefore, further large-scale research is needed to investigate this issue.

## Materials and methods

### Sample and strain collection

A total of 160 samples were collected from DFI patients who met the inclusion and exclusion criteria. The samples were collected from Nanfang Hospital of Southern Medical University (Guangzhou, China) between December 2020 and December 2021. These samples were taken from the wound tissue obtained by removing necrotic tissue from the feet of DFIs patients. The clinical information of the 522 DFIs patients involved in the above-mentioned collection of the 160 samples was also collected at this hospital.

Inclusion criteria: clinical diagnosis of diabetic foot, combined with foot ulceration or deep tissue destruction, accompanied by local redness, swelling, obvious secretion, and a leukocyte count of more than 12,000/mm^3 in the routine blood count. Additionally, changes in inflammatory serum biomarkers will be considered, with a requirement of C-reactive protein (CRP) levels of more than 5 mg/L as an auxiliary measure to determine the diagnosis. Exclusion criteria: clinical diagnosis of diabetic foot without any signs or symptoms of systemic or localized infection [31].

The 19 *S. aureus* strains used in this experiment, including 4 MSSA, 15 MRSA, and 7 other types of bacteria, were provided by the Clinical Laboratory Department. The collection of the patients’ clinical information and samples was reviewed and approved by the Ethics Committee of Nanfang Hospital (NFEC-202012-K6) and registered on ClinicalTrials.gov (NCT04916457). Informed consent forms were signed by patients or their surrogates.

### Rapid DNA Extraction

Samples were diluted in 1 mL of sterilized saline and shaken for one minute. Then, 100 µl of eluate was transferred to a new nuclease-free 200 µl PCR tube and centrifuged for five minutes using a mini centrifuge. The pellet was resuspended in 50 µl of lysis buffer (Takara, 9164) and incubated at 80 °C for 15 min. The supernatant was used as a DNA template for LAMP detection and PCR analysis.

### Enrichment and monoclonal culture

200 µl of diluted samples were transferred to 3 mL of LB medium containing 7.5% sodium chloride and incubated at 35 °C and 220 rpm for 20 h. Products were then inoculated into blood agar plates (Autobio) and cultured at 35 °C for 24 h. Based on the color, shape, and size of the strain, a monoclone was selected and transferred to a new blood agar plate for an additional 24 h. The selected strain was then sent to the laboratory for mass spectrometry analysis to identify the *S. aureus* strain.

### LAMP detection

The LAMP method relies on auto-cycling strand displacement DNA synthesis that is performed by a DNA polymerase with high strand displacement activity and a set of six LAMP primers (four core and two loop) [32, 33]. LAMP primers were designed for the *S. aureus* genome using the online PrimerExplorer V5 website (http://primerexplorer.jp/lampv5e/). Sequence information on the primers is shown in the Supplement table. The LAMP reaction solution contained 2.5 µl of DNA template, 0.24 µM each of F3 and B3, 0.48 µM each of LF and LB, 1.92 µM each of FIP and BIP, 6 mM MgSO_4_, 1.4 mM dNTP (CoWin Biosciences, CW0941), 8 U Bst 2.0 Warmstart DNA polymerase (NEB, M0538S), 1 × isothermal amplification buffer, 0.2 M betaine (Sigma, B0300), and 120 µM hydroxynaphthol blue (Macklin, H810857). The reaction mixtures were incubated at 60 °C for 60 min and observed by eye.

### PCR

PCR amplification was performed according to the manufacturer’s instructions, and the reactions contained 2.5 µl of DNA template, 10 µM each of F3 and B3 primers, and 25 µl of 2 × ES Taq MasterMix (CoWin Biosciences, CW0609 M). The amplification products were detected using 2% agarose gel electrophoresis, and the band was analyzed under UV illumination.

### Cefoxitin antimicrobial susceptibility testing

The cefoxitin disc diffusion test was performed following the Clinical and Laboratory Standards Institute (CLSI) guidelines for the identification of MRSA. The standard for determining whether the strain is resistant or not is based on the instructions for the cefoxitin test discs (thermo, DD0026B). Briefly, monoclonal cultures were inoculated into 3 ml of broth medium and incubated at 35 °C and 220 rpm for 6 h. The turbidity of the bacterial solution was adjusted to 0.5 McFarland (MCF) using sterile saline and then spread onto the surface of the plate. Discs were applied to the plate within 15 min and then incubated at 35 °C for 24 h.

### Statistical analysis

Statistical analysis was performed using SPSS version 26.0 (IBM Corp., Armonk, NY, USA). Continuous variables were expressed as the median and interquartile range (25th, 75th percentile) and were compared using the Mann–Whitney U test. Categorical variables were expressed as frequencies and percentages and were compared with the chi-squared test. All tests were two-sided, and results with a *p*-value < 0.05 were considered statistically significant.

### Supplementary Information

**Additional file 1: Supplement Table.** LAMP primers used in this study.

**Additional file 2.**

## Data Availability

The data presented in this study is available upon request from the corresponding author.

## Abbreviations

*S. **aureus*: *Staphylococcus aureus*
DFIs: Diabetic foot infections
LAMP: Loop-mediated isothermal amplification
MRSA: Methicillin-resistant *Staphylococcus aureus*
MSSA: Methicillin-susceptible *Staphylococcus aureus*
DFUs: Diabetic foot ulcers
PBP2a: Penicillin binding protein
MRSCN: Coagulase-negative methicillin-resistant staphylococci
SCCmec: Staphylococcal chromosomal cassettes mec
