# Supplementary material for: Prevalence of S. aureus and/or MRSA in hospitalized patients with diabetic foot and establishment of LAMP methods for rapid detection of the SCCmec gene
Source: BMC Microbiol. 2024 Jan 26;24:36. doi: 10.1186/s12866-024-03196-6 (PMC10811927; doi:10.1186/s12866-024-03196-6)
Supplement: Supplementary file 1 — Additional file 1: Supplement Table. LAMP primers used in this study. [file 12866_2024_3196_MOESM1_ESM.docx]

supplement Table. LAMP primers used in this study

| Target | Primers | Sequence (5'-3') | Positions |
| --- | --- | --- | --- |
| femA | FIP-1 | ACCACCAGCATAATAAACAACTTCA  AATTACCTATCTCTGCTGGTTTC | F1c: 969-993  F2: 929-951 |
|  | BIP-1 | GCATTCCGTCATTTTGCCGG  TAACGGTCAATGCCATGA | B1c: 1003-1022  B2: 1065-1082 |
|  | F3-1 | AACGTCTACAAGAAGAACATG | 902-922 |
|  | B3-1 | TGTAAATTTACCACTAACACCATAG | 1089-1113 |
|  | LF-1 | AATGGATTGATAAAGAA | 952-968 |
|  | LB-1 | TGCAGTGCAATGGGAAATGA | 1029-1048 |
| mecA | FIP-2 | TGAAGGTGTGCTTACAAGTGCTAAT  CAACATGAAAAATGATTATGGCTC | F1c: 1071-1095  F2: 1014-1037 |
|  | BIP-2 | TGACGTCTATCCATTTATGTATGGC  AGGTTCTTTTTTATCTTCGGTTA | B1c: 1098-1022  B2: 1148-1170 |
|  | F3-2 | TGATGCTAAAGTTCAAAAGAGT | 984-1005 |
|  | B3-2 | GTAATCTGGAACTTGTTGAGC | 1173-1193 |
|  | LF-2 | TTGAGGGTGGATAGCAGTACCT | 1038-1059 |
|  | LB-2 | TGAGTAACGAAGAATAT | 1124-1140 |
| SCCmec type II | FIP-3 | ACCGCATCATTTATGATATGCTTCT  CGGGTTGTGTTAATTGAGC | F1c: Orfx 462-480 and  SCCmec type II 1-6  F2: orfx 412-430 |
|  | BIP-3 | TCAGCCGCTTCATAAAGGGAT  AAGCTTCTTAAAAACATAACAGC | B1c: SCCmec type II 11-31  B2: SCCmec type II 69-91 |
|  | F3-3 | AAAATGACATTCCCACATCAA | Orfx 385-405 |
|  | B3-3 | TGTCAAAAATCATGAACCTCAT | SCCmec type II 102-123 |
|  | LF-3 | CCACGCATAATCTTAAATGC | Orfx 442-461 |
|  | LB-3 | TGTATCAGAACATATGAGGT | SCCmec type II 38-57 |
