# Supplementary material for: Prevalence of S. aureus and/or MRSA in hospitalized patients with diabetic foot and establishment of LAMP methods for rapid detection of the SCCmec gene
Source: BMC Microbiol. 2024 Jan 26;24:36. doi: 10.1186/s12866-024-03196-6 (PMC10811927; doi:10.1186/s12866-024-03196-6)

Figure 1C Images showing full length membranes

These images are the original, unprocessed versions and contain electrophoresis results of other samples who are not mentioned in this article.


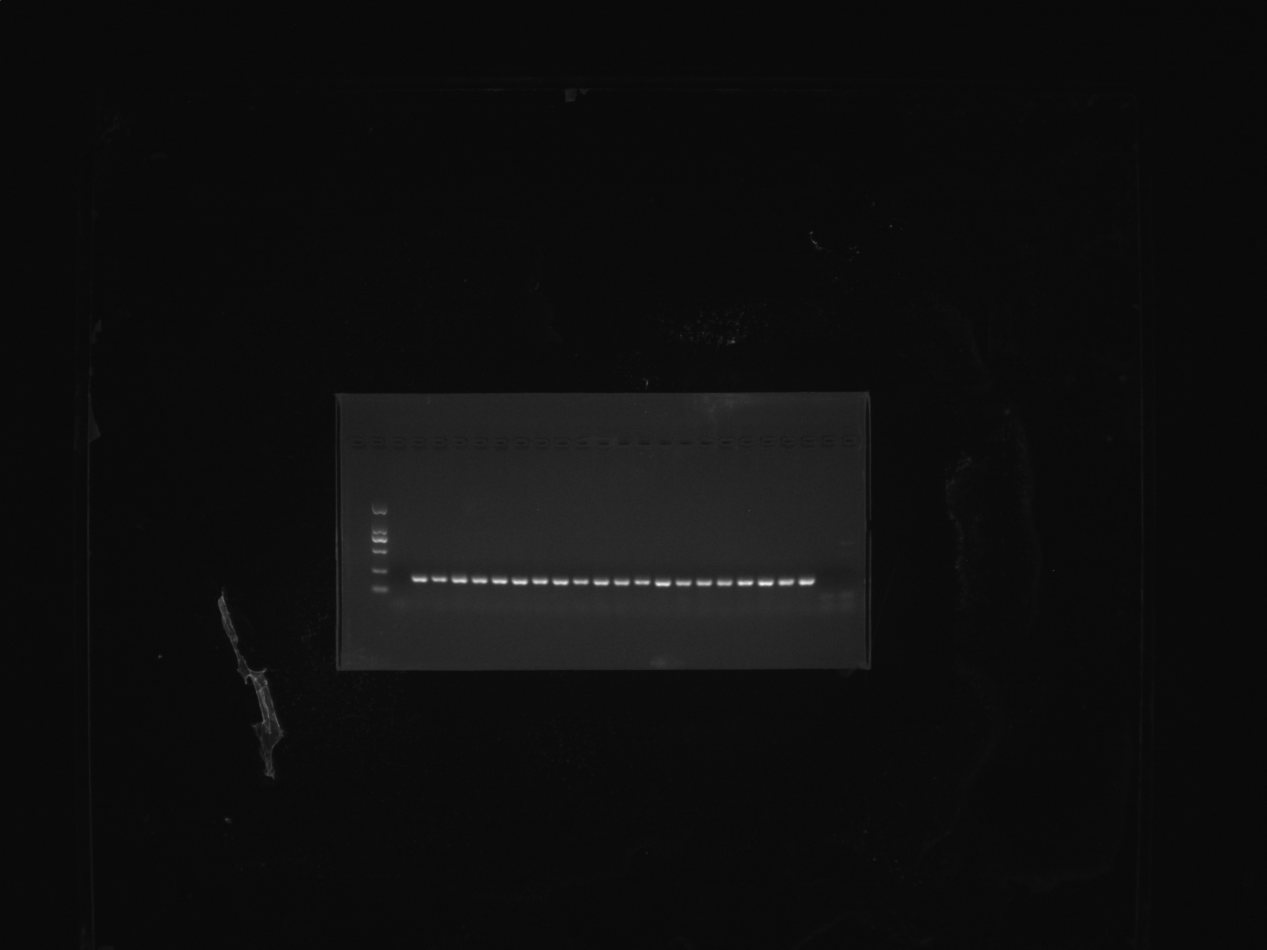


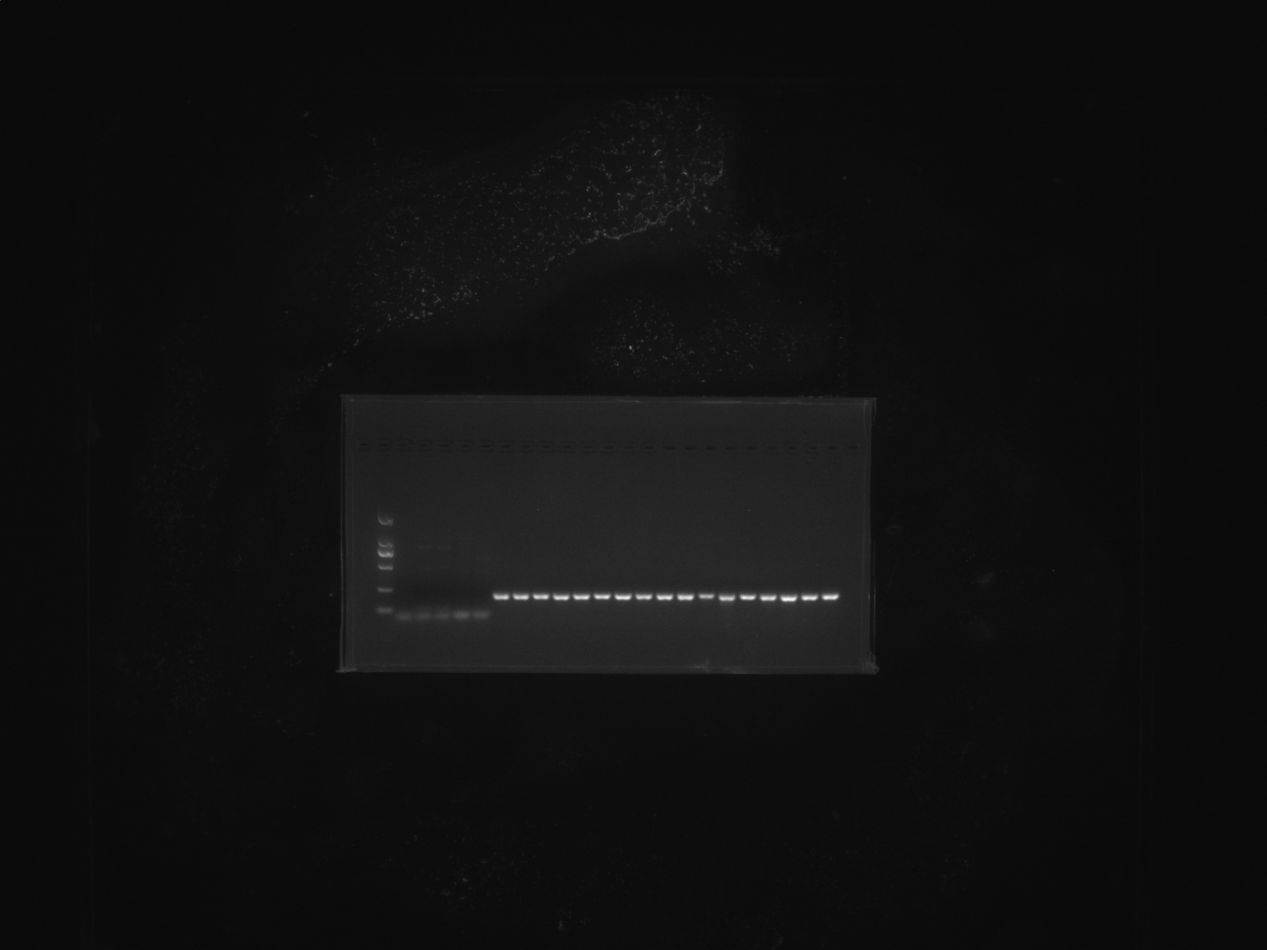

Supplement: Supplementary file 2 — Additional file 2. [file 12866_2024_3196_MOESM2_ESM.docx]
